# Multi-Omic Analyses Reveal Bifidogenic Effect and Metabolomic Shifts in Healthy Human Cohort Supplemented With a Prebiotic Dietary Fiber Blend

**DOI:** 10.3389/fnut.2022.908534

**Published:** 2022-06-17

**Authors:** Jea Woo Kang, Xinyu Tang, Charles J. Walton, Mark J. Brown, Rachel A. Brewer, Rolando L. Maddela, Jack Jingyuan Zheng, Joanne K. Agus, Angela M. Zivkovic

**Affiliations:** ^1^Department of Nutrition, University of California, Davis, Davis, CA, United States; ^2^USANA Health Sciences, Inc., Salt Lake City, UT, United States

**Keywords:** prebiotic, gut microbiome, bifidobacteria, indolepropionate, cholines

## Abstract

Dietary fiber, a nutrient derived mainly from whole grains, vegetables, fruits, and legumes, is known to confer a number of health benefits, yet most Americans consume less than half of the daily recommended amount. Convenience and affordability are key factors determining the ability of individuals to incorporate fiber-rich foods into their diet, and many Americans struggle to access, afford, and prepare foods rich in fiber. The objective of this clinical study was to test the changes in microbial community composition, human metabolomics, and general health markers of a convenient, easy to use prebiotic supplement in generally healthy young participants consuming a diet low in fiber. Twenty healthy adults participated in this randomized, placebo-controlled, double-blind, crossover study which was registered at clinicaltrials.gov as NCT03785860. During the study participants consumed 12 g of a prebiotic fiber supplement and 12 g of placebo daily as a powder mixed with water as part of their habitual diet in randomized order for 4 weeks, with a 4-week washout between treatment arms. Fecal microbial DNA was extracted and sequenced by shallow shotgun sequencing on an Illumina NovaSeq. Plasma metabolites were detected using liquid chromatography–mass spectrometry with untargeted analysis. The phylum Actinobacteria, genus *Bifidobacterium*, and several *Bifidobacterium* species (*B. bifidum, B. adolescentis, B. breve, B. catenulatum*, and *B. longum)* significantly increased after prebiotic supplementation when compared to the placebo. The abundance of genes associated with the utilization of the prebiotic fiber ingredients (*sacA, xfp, xpk*) and the production of acetate (*poxB, ackA*) significantly changed with prebiotic supplementation. Additionally, the abundance of genes associated with the prebiotic utilization (*xfp, xpk*), acetate production (*ackA*), and choline to betaine oxidation (*gbsB*) were significantly correlated with changes in the abundance of the genus *Bifidobacterium* in the prebiotic group. Plasma concentrations of the bacterially produced metabolite indolepropionate significantly increased. The results of this study demonstrate that an easy to consume, low dose (12 g) of a prebiotic powder taken daily increases the abundance of beneficial bifidobacteria and the production of health-promoting bacteria-derived metabolites in healthy individuals with a habitual low-fiber diet.

**Clinical Trial Registration:**

www.clinicaltrials.gov/, identifier: NCT03785860

## Introduction

Many Americans consume diets that are deficient in fiber for reasons that range in nature from financial constraints to personal taste preferences. Dietary fibers are polymers of monosaccharides which are resistant to human digestive enzymes, and have been shown to have beneficial effects on metabolism including improvements in glucose and insulin levels, and reduction of blood cholesterol concentrations (1–3). Dietary fiber intake is also associated with increases in the overall diversity of the gut microbiome (4) and in the abundance of beneficial microbial taxa, i.e., *Bifidobacterium* (5). The presence of bifidobacteria in the gut microbiome is associated with multiple health benefits including short-chain fatty acid (SCFA) production (4, 6) and improved gut barrier functionality (7), which in turn are linked with reduced inflammation (8), reduced concentrations of circulating lipopolysaccharide (9, 10), and improved gut immune function (11, 12). Despite the fact that the daily consumption of dietary fiber is associated with beneficial health effects, the average daily intake of dietary fiber in the U.S. is only ~16 g; less than half of the amount recommended by the USDA dietary guidelines (13). There are many practical obstacles that individuals face when considering lifestyle changes that are necessary to increase the intake of dietary fiber. Many Americans do not have access to fresh produce, do not have the time or the knowledge to prepare meals containing fiber-rich foods, or simply cannot afford to do so (14–17). Thus, practical solutions, like fiber supplements, are needed to increase fiber intake in individuals who are consuming low-fiber diets, or who are not able to implement the necessary lifestyle changes that increase fiber intake through whole foods.

A prebiotic is defined as a substrate that is selectively utilized by host microorganisms conferring a health benefit (18). Prebiotics, including inulin, fructooligosaccharides, galactooligosaccharides, and resistant starch, are known to increase the abundance of beneficial gut microbiota, particularly bifidobacteria (19–22). The increased abundance of saccharolytic microbes is in turn associated with enhanced production of microbially derived secondary metabolites that improve overall gut and metabolic health (23). The beneficial effects of SCFA on gut barrier integrity, gut immune function, and overall metabolism have been extensively documented (23–30). Other microbially produced metabolites with potential beneficial or deleterious effects on human health have been identified utilizing targeted and untargeted metabolomic approaches. For example, the metabolite indolepropionate (IPA) has been associated with beneficial health effects (31–33), whereas the metabolite trimethylamine-N-oxide (TMAO) has been linked with an increased risk for cardiovascular disease (34, 35).

In this study, we tested the effects of a prebiotic dietary fiber supplement formulation on the gut microbiome and human metabolome in 20 participants using a randomized, double-blind, placebo-controlled, crossover study design. We hypothesized that 4 weeks of daily supplementation with a prebiotic fiber blend will: increase the abundance of bifidobacteria and the abundance of gut microbial genes associated with bacterial utilization of the prebiotic substrate, alter fecal SCFA concentrations and gut microbial genes related to the production of SCFA, modify plasma metabolites, specifically, increase IPA and decrease TMAO, and alter cardiometabolic profiles.

## Materials and Methods

### Participants

Twenty healthy men and women aged 18–45 y, BMI of 23.0–32.0 kg/m^2^, with a habitual diet low in fiber (<15 g/day) were enrolled at the Ragle Human Nutrition Center, University of California (UC), Davis. Recruitment began in April of 2019 and the study completion date was December of 2019. Exclusion criteria included for screening were: smoking, having anemia and difficulty with blood draws, use of probiotic or prebiotic formulations within 6 weeks of the study start date, use of antibiotics within 6 months prior to study commencement, use of medication such as statins, blood pressure medications, and other prescription medications, pregnancy, use of hormonal birth control in the last 6 months or plans to change or start use of hormonal birth control during the study period, allergies to any placebo or prebiotic ingredients, presence of illness (flu/cold in the last 2 weeks), presence of documented chronic diseases, presence of intestinal diseases (irritable bowel syndrome, celiac disease, or any inflammatory bowel disease including Crohn's disease and/or ulcerative colitis), presence of any immunosuppression symptoms at any point during the study or study enrollment, consumption of >1 alcoholic drink/day or frequent binge drinking (>3 alcoholic drinks in one episode) of >1 day/month, plans to change or recent significant changes in lifestyle (e.g., diet, exercise routine, or major travel), recent weight fluctuations (>10% in the last 6 months), regular use of over-the-counter pain medications (>1/week), use of prescription lipid medications or other supplements known to alter lipoprotein metabolism such as isoflavones, recent medical procedure such as surgery within the last 6 months, and any changes in the above during the course of the study.

**Figure 1:**
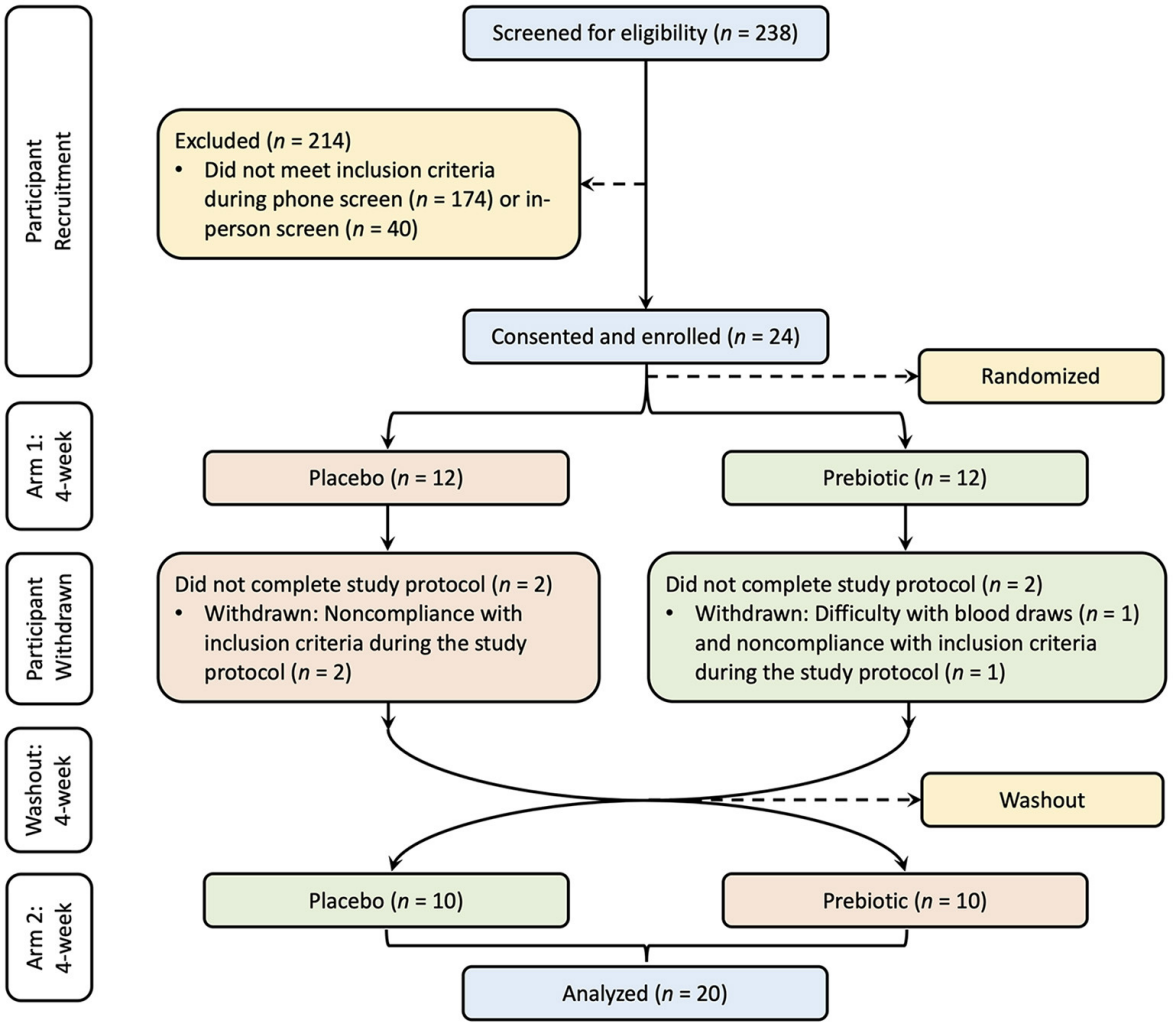
Study CONSORT diagram. Participants were recruited with screening, consent, and enrollment process. The treatment order was randomized into two groups, one group (red) supplemented with the placebo and the other group (green) supplemented with the prebiotic followed by a washout period and crossover to the other treatment for each group.

The sample size was determined based on a previous study which included 25 healthy participants supplemented with an inulin-type fructan-diet, in which a significant increase in bifidobacteria was observed (36). The study was approved by the Institutional Review Board of UC Davis. Written consent was provided by all participants prior to entry into the protocol. One participant was withdrawn due to difficulty with blood draws, three participants were withdrawn due to non-compliance with inclusion criteria, and 20 participants (10 male and 10 female) completed the study (Figure 1). This clinical trial was registered at www.clinicaltrials.gov/ as NCT03785860.

### Study Design

The study was a randomized, double-blind, placebo-controlled, crossover design. All participants consumed a prebiotic fiber supplement and a matching placebo for a period of 4 weeks each, with a 4-week washout between intervention arms in random order. Twelve participants were randomly assigned to the prebiotic blend as their first intervention arm and 12 were assigned to the placebo blend as their first intervention arm. Four participants were withdrawn during the first intervention arm. After completion of the first intervention arm and the 4-week washout in which participants maintained their habitual diet without changing their regular lifestyle, the participants switched to the other intervention arm. Twenty subjects completed the study and were included in the analysis.

The prebiotic fiber blend used in this study was a dry mixture of fructooligosaccharides, resistant starch, sugarcane fiber, inulin, gum arabic, xanthan gum, and apple, raspberry, and blueberry fruit powders provided in single use packets of 12 g each. The placebo packets, also 12 g each, contained rice flour, xanthan gum and grape and plum fruit powders to match the prebiotic supplement in taste and appearance. Participants were provided the coded powder packet and instructed to mix with water for consumption. Participants were asked to record daily consumption of the powder packet and to bring back the empty packet for verification of compliance with study protocols.

Using 24-h diet records, participants recorded their diet for 3 days prior to each study visit at 2-week intervals for the duration of the study. The diet was patternized each week, meaning participants were asked to consume the same meals and foods for the 3 days prior to each test day without significantly changing their usual diet. This was done to stabilize the background diet during the days leading up to fecal sample collection, since it has been observed that dietary fluctuations over the preceding 3–4 days can significantly influence the gut microbiome (37, 38). Diet records were analyzed using the nutrition software, Food Processor SQL (version 11.7; ESHA).

Questionnaires and anthropometric measurement data were collected at each visit. The SF-12^®^ Health questionnaire was collected to track the general health status of participants during the study intervention. Bowel movement type and frequency, which was rated by the participants throughout the study, was evaluated using the Bristol stool scale (39) and modified bowel movement questionnaire (40). Height was measured with a wall-mounted stadiometer (Ayrton Corp.) and body weight was measured with a calibrated electronic scale (Scale-Tronix; Welch Allyn). Blood pressure was measured with an automated sphygmomanometer (OxiMax; Welch Allyn) in a seated position. All measurements were performed in triplicate and the average was used for anthropometric measurement data.

### Blood Sample Collection and Analysis of Cardiometabolic Profiles

Whole blood samples were collected after a 12-h overnight fast at the beginning and end of each intervention arm. Blood samples were collected in EDTA, SST, and PST tubes (Becton Dickinson) *via* venipuncture by a certified phlebotomist. The samples collected in EDTA tubes were immediately centrifuged (Sorvall-Legend RT) at 1,500 × g, 4°C for 10 min. Plasma samples were aliquoted immediately after centrifugation and stored at −80°C until further analyses. Blood samples collected in PST tubes were immediately centrifuged at 1,300 × g, 4°C for 10 min. The samples in SST tube were allowed to sit for 30 min for the blood to clot and then were centrifuged at 1,300 × g, 4°C for 10 min. Samples collected in PST and SST tubes were sent to UC Davis Medical Center Pathology Lab for analyses of glucose, lipid profiles, and insulin. Glucose was analyzed using the glucose oxidase method measuring absorbance at 652 nm after the peroxide catalyzed reaction (41). Insulin was analyzed with the Abbott Architect i1000 chemiluminescent microparticle immunoassay (42). A lipid panel [total cholesterol (TC), triacylglycerols (TG), HDL-cholesterol and calculated LDL-cholesterol] analysis was performed using a clinical analyzer (DXC 800; Beckman Coulter). TC, TG, and HDL-cholesterol were directly measured, and LDL-cholesterol was calculated using the Friedewald equation (43).

### Stool Sample Collection

Fecal samples were self-collected by study participants. Prior to each stool sample collection day, participants were given stool collection kits consisting of a preservative-filled fecal collection tube (OMNIgeneGUT, OMR-200, DNA Genotek, Ottawa, ON, Canada), fecal collection sheet (Easy Sampler, EU version, GP Medical Devices, Holstebro, Denmark), and sanitizing kit. Trained study personnel educated participants on the stool collection protocol in person prior to the start of the study. Written instructions were also provided in the collection kits to ensure proper collection protocols were followed and to minimize sample contamination and deterioration. Participants were instructed to immediately transfer collected stool samples to a portable cooler packed with frozen icepacks and bring in the collected samples as soon as possible within 24 h of sample collection, including a transfer time of 4 h at most. Upon arrival to the laboratory, the samples were aliquoted into Eppendorf tubes and immediately stored at −80°C until analysis.

### DNA Extraction, Sequencing, Preprocessing, and Assembly

Stool samples were processed at Diversigen (Houston, TX, USA) and shallow shotgun metagenomic sequencing was performed following DNA extraction. Briefly, DNA extraction from raw stool samples was performed with the DNeasy PowerSoil Pro Kit (Qiagen) automated for high throughput on the QiaCube HT (Qiagen) using Powerbead Pro (Qiagen) plates with 0.5 mm and 0.1 mm ceramic beads. Samples were quantified with Quant-iT Picogreen dsDNA Assay (Invitrogen). Libraries were prepared with a procedure adapted from the Nextera Library Prep kit (Illumina). Libraries were sequenced on an Illumina NovaSeq using single-end 1 × 100 cycles (Illumina). DNA sequences were filtered for low quality (Q-Score <30) and length (<50), and adapter sequences were trimmed using cutadapt (v1.15) (44). The sequences for each sample were assembled into contigs using SPAdes (v3.11.0) (45). The quality of assemblies for contigs >1,000 bases in length were assessed using QUAST (v4.5) (46).

### Gene Annotation and Taxonomy Inference

Prokka (v1.12) (47) was used to annotate genes for each strain using the contigs > 1,000 bases as described above. Annotation files were parsed and combined to make gene content comparison tables. For taxonomy inference, trimmed and quality filtered sequences were aligned to every reference sequence in CoreBiome's Venti database at 97% identity using fully gapped alignment with BURST (v1.00) (48). Ties were broken by minimizing the overall number of unique gene, Operational Taxonomic Units (OTUs), hits. For taxonomy assignment, each input sequence was assigned the lowest common ancestor that was consistent across at least 80% of all reference sequences tied for best hits. The three most abundant taxa were reported for each strain.

Filtered taxonomy tables were generated. Briefly, samples with fewer than 10,000 sequences were discarded. OTUs accounting for less than one millionth of all strain-level markers and those with <0.01% of their unique genome regions covered (and <0.1% of the whole genome) at the species level were discarded. The number of counts for each OTU was normalized to the OTU's genome length for downstream analysis. The relative abundance of each sample was calculated by dividing OTU counts of each sample from the sum OTU counts of total sample using phyloseq (v1.36.0) (49).

### Functional Annotation and Profiling

For functional annotation and profiling, Kyoto Encyclopedia of Genes and Genomes Orthology groups (KEGG KOs) were observed directly using alignment at 97% identity against a gene database derived from the strain database used above (Diversigen Venti database). The KEGG Orthology group table was filtered to the same subset of samples as the filtered taxonomic tables and used for downstream analysis.

### Short-Chain Fatty Acid Analysis

Stool samples were processed at Microbiome Insights (University of British Columbia Vancouver, BC, Canada). Briefly, the SCFA extraction procedure is similar to that of Zhao et al. (50). Material was resuspended in MilliQ-grade H_2_O, and homogenized using MP Bio FastPrep, for 1 min at 4.0 m/s. Fecal suspensions were acidified with 5 M HCl to a final pH of 2.0. Acidified fecal suspensions were incubated and centrifuged at 10,000 rpm to separate the supernatant. Fecal supernatants were spiked with 2-ethylbutyric acid for a final concentration of 1 mM. Extracted SCFA supernatants were stored in 2-mL GC vials with glass inserts. Short-chain fatty acids were detected using gas chromatography (Thermo Trace 1310), coupled to a flame ionization detector (Thermo). The column used for SCFA detection was Thermo TG-WAXMS A GC Column (30 m, 0.32 mm, 0.25 μm) with a flow rate of 6.0 mL/min. The flame ionization detector was set to 240°C with controlled amounts of hydrogen: 35.0 mL/min, air: 350.0 mL/min, and makeup gas (Nitrogen): 40.0 mL/min. Short-chain fatty acid standards were acetic acid, propionic acid, isobutyric acid, butyric acid, isovaleric acid, valeric acid, and hexanoic acid from Sigma Aldrich.

### Metabolomics

Untargeted metabolomic analysis was performed on the plasma samples at Metabolon (Morrisville, NC, USA) as previously described (51). All samples were maintained at −80°C until processing. Briefly, individual samples were subjected to methanol extraction then split into aliquots for analysis by ultra-high performance liquid chromatography-mass spectrometry (UHPLC-MS). The global biochemical profiling analysis comprised of four unique arms consisting of reverse phase chromatography positive ionization methods optimized for hydrophilic compounds (LC/MS Pos Polar) and hydrophobic compounds (LC/MS Pos Lipid), reverse phase chromatography with negative ionization conditions (LC/MS Neg), as well as a hydrophilic interaction liquid chromatography (HILIC) method coupled to positive and negative electrospray ionization modes (LC/MS Polar) (52). All the methods were alternated between full scan MS and data dependent MS^*n*^ scans. The scan range varied slightly between methods but generally covered 70–1,000 m/z. Metabolites were identified by automated comparison of the ion features in the experimental samples to a reference library of chemical standard entries from Metabolon that included retention time, molecular weight (*m*/*z*), preferred adducts, and in-source fragments as well as associated MS spectra and curated by visual inspection for quality control using software developed at Metabolon. Identification of known chemical entities was based on comparison to metabolomic library entries of purified standards (53).

### Statistical Analysis

Differential expression analyses of OTU counts and gene counts were performed with the package DESeq2 (54) in R version 4.0.2 (R Foundation for Statistical Computing, Vienna, Austria), which is based on a negative binomial model extended with Wald methods. To identify the OTU and gene differences between treatments over time, the count data was fitted into a generalized linear model with a design matrix: count ~ treatment ^*^ timepoint + subject. OTUs and genes that responded differently to the treatment relative to placebo at either timepoint were tested using the Wald test in the DESeq2 package (54). The primary outcome of the study was the change in the OTU counts of *Bifidobacterium* associated with the prebiotic supplement compared to the placebo. Therefore, we performed the differential expression analysis at the genus level. The secondary outcomes of the study were changes in other microbe abundances as well as gene counts, cardiometabolic profiles, SCFA concentrations, anthropometric measurements, and plasma metabolomic profiles. Specifically, we hypothesized that the gene counts of *sacA, xfp, xpk, poxB, ackA*, and *buk* genes would be altered on the prebiotic arm. Also, we hypothesized that the concentrations of IPA would increase, whereas TMAO would decrease on the prebiotic arm. The same data analysis pipeline was applied to gene count data. Linear mixed models with the previously mentioned design matrix were used to determine changes in cardiometabolic profiles, SCFA concentration, anthropometric measurements, and plasma metabolomic profiles with the R package limma (55). The Shapiro-Wilk normality test was performed prior to downstream analysis to check for variable normality. Log transformations were performed if variables did not fit a normal distribution. For any exploratory analyses, the Benjamini–Hochberg false discovery rate was calculated to adjust for multiple testing. The phyloseq R package was used to calculate the microbiome diversities (49). Kendall's correlation was performed to study novel relationships between changes in OTU counts of the genus *Bifidobacterium* and changes in gene counts of bacterial genes as well as metabolomic profiles. KEGG pathway based gene set enrichment analysis (GSEA) was performed and visualized using ClusterProfiler package in R (56) to test the effect of prebiotic treatment on metabolic pathways which include genes enriched by the treatment against the placebo. Partial least-squares discriminant analysis (PLS-DA) was performed to investigate the effect of the prebiotic treatment on overall metabolomic profiles using the pls and caret packages in R (57, 58).

## Results

### Anthropometrics, Cardiometabolic Profiles, and Diet Records

Participant baseline characteristics and their cardiometabolic profiles pre and post the prebiotic and placebo arms are summarized in Table 1. No significant changes were found in anthropometric measurements. Cardiometabolic profiles showed no significant differences between prebiotic and placebo groups. Also, there were no significant changes in nutrient intake at any time point (Table 2). No significant changes were observed for bowel movement type and frequency during the study period (data not shown).

**Table 1:** Participant anthropometric and cardiometabolic characteristics pre- and post-treatment on placebo and prebiotic fiber^*^.

**Variable**	**Placebo**		**Prebiotic**		***P*-value**
	**Pre**	**Post**	**Pre**	**Post**	
Age, y	27.1 ± 6.1	27.1 ± 6.1	27.1 ± 6.1	27.1 ± 6.1	NA
Weight, kg	74.8 ± 10.4	74.8 ± 10.1	75.5 ± 10.2	74.9 ± 10.7	0.29
Height, cm	169.3 ± 9.0	169.3 ± 9.3	169.3 ± 9.1	169.4 ± 9.2	0.84
BMI, kg/m^2^	26.1 ± 2.9	26.1 ± 2.9	26.3 ± 2.9	26.1 ± 2.8	0.17
SBP, mmHg	113.5 ± 7.3	112.8 ± 7.7	112.8 ± 6.5	113.9 ± 6.7	0.36
DBP, mmHg	74.2 ± 4.7	73.7 ± 5.0	74.1 ± 5.4	74.4 ± 5.5	0.44
Fasting glucose, mg/dL	83.6 ± 6.3	85.8 ± 7.7	86.2 ± 7.0	85.3 ± 7.1	0.29
Fasting insulin, μIU/mL	6.3 ± 3.3	6.9 ± 3.8	6.2 ± 3.9	6.1 ± 3.2	0.42
TG, mg/dL	73.1 ± 40.8	64.0 ± 31.6	69.4 ± 37.1	69.0 ± 29.9	0.38
TC, mg/dL	176.2 ± 23.6	175.1 ± 221.3	176.1 ± 25.9	172.3 ± 25.1	0.61
LDL cholesterol, mg/dL	109.2 ± 21.9	107.7 ± 19.9	109.4 ± 21.7	106.2 ± 22.1	0.71
HDL cholesterol, mg/dL	52.4 ± 12.1	54.5 ± 15.0	52.8 ± 12.2	52.4 ± 11.9	0.12
TC:HDL cholesterol	3.5 ± 0.8	3.4 ± 0.8	3.4 ± 0.6	3.4 ± 0.7	0.55
Non-HDL cholesterol, mg/dL	123.8 ± 22.8	120.6 ± 21.6	123.3 ± 22.9	120.0 ± 23.1	1.00

* *Data are shown as means ± SDs. Changes on pre- and post-treatment with placebo or prebiotic were compared with a linear mixed model (n = 20)*.

*SBP, systolic blood pressure; DBP, diastolic blood pressure; TG, triacylglycerol; TC, total cholesterol*.

**Table 2:** The composition of the background diet of participants pre- and post-treatment with placebo and prebiotic fiber^*^.

**Variable**	**Placebo**		**Prebiotic**		***P*-value**
	**Pre**	**Post**	**Pre**	**Post**	
Total kcal	2,182.2 ± 634.7	1,866.7 ± 592.9	1,871.2 ± 655.9	2,008.5 ± 811.4	0.09
Carbohydrate, g	245.1 ± 96.1	215.2 ± 121.7	212.5 ± 85.3	220.9 ± 129.4	0.36
Protein, g	101.9 ± 56.0	84.8 ± 30.8	85.9 ± 44.5	89.8 ± 39.6	0.13
Fat, g	89.5 ± 38.9	75.1 ± 28.3	76.0 ± 38.5	84.3 ± 42.5	0.16
Total dietary fiber, g^**^	18.0 ± 7.9	14.4 ± 9.5	13.5 ± 6.4	13.2 ± 7.7	0.21

* *Data are shown as means ± SDs. Changes on pre- and post-treatment with placebo or prebiotic were compared with a linear mixed model (n = 20)*.

** *Prebiotic supplement was not included in the background dietary intake of total dietary fiber*.

### Gut Microbial Composition

The overall gut microbial diversity was calculated using the Shannon diversity index to measure alpha diversity of microbiome species in samples and the Bray–Curtis dissimilarity index to evaluate beta diversity of species difference between the placebo and prebiotic intervention arms. There were no significant changes in microbial diversity after the prebiotic intervention compared to the placebo (Supplementary Figures 1A–C).

**Figure 2:**
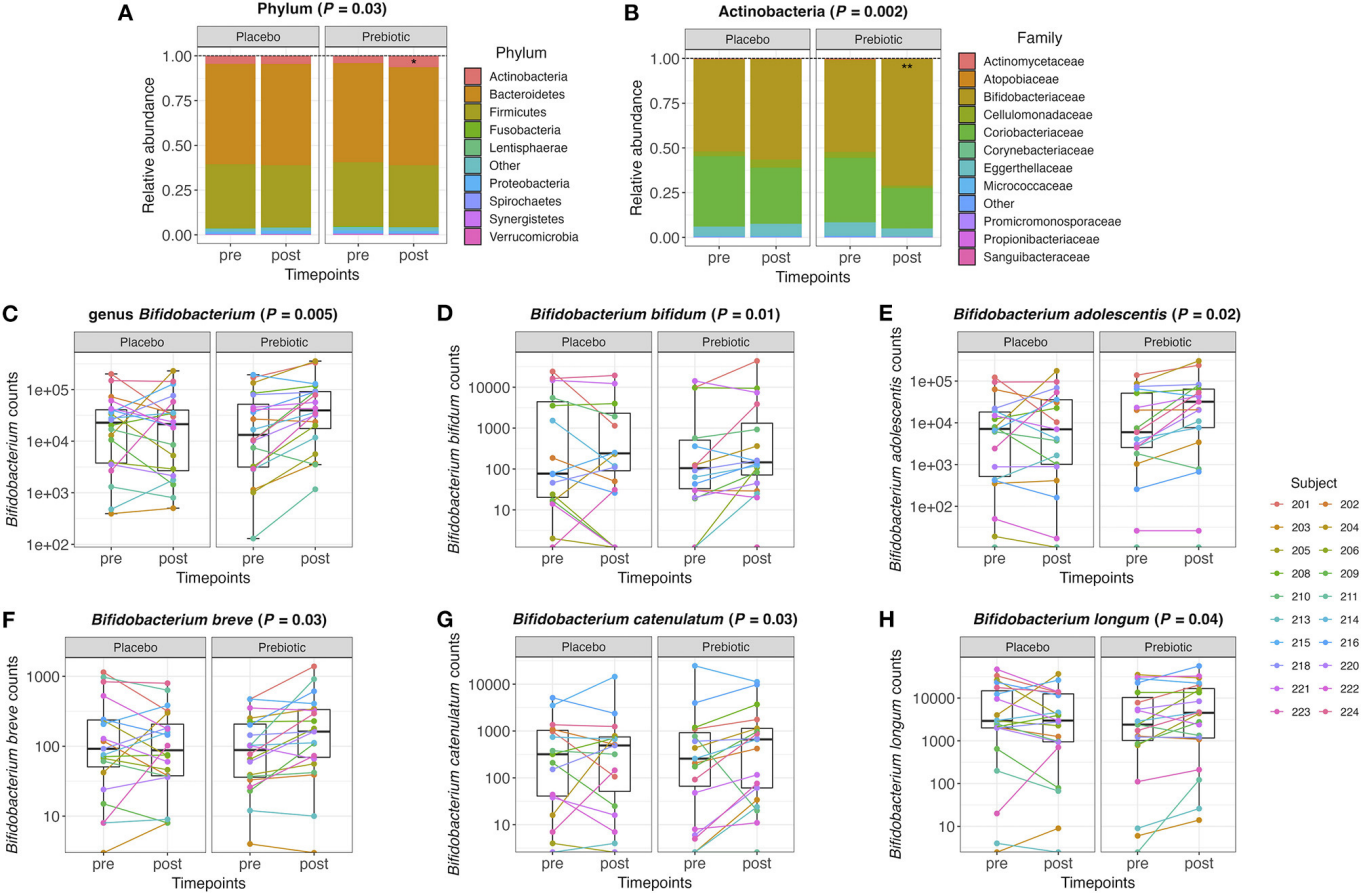
**(A)** Relative abundance of the gut microbiome at the phylum level and **(B)** the family level within the phylum Actinobacteria pre- and post-treatment with placebo or prebiotic. **(C)** Box plots of the genus *Bifidobacterium* counts pre- and post-treatment with placebo or prebiotic. **(D–H)** Box plots of *Bifidobacterium* species counts pre- and post-treatment with placebo or prebiotic (*P <0.05, **P < 0.01).

Further analyses on relative abundance of all gut microbiota in fecal samples were performed from phylum to species level. The relative abundance of the phylum Actinobacteria significantly increased after the prebiotic (*P* = 0.03) compared to the placebo arm (Figure 2A). Under the phylum Actinobacteria, the family Bifidobacteriaceae significantly increased after the prebiotic (*P* = 0.002) compared to the placebo arm (Figure 2B). *Bifidobacterium* counts were significantly increased after the prebiotic treatment (*P* = 0.005) compared to the placebo treatment (Figure 2C). In addition, several species belonging to the genus *Bifidobacterium* increased in the prebiotic arm compared to the placebo arm (Figures 2D–H), including *Bifidobacterium bifidum* (*P* = 0.01, unadjusted), *Bifidobacterium adolescentis* (*P* = 0.02, unadjusted), *Bifidobacterium breve* (*P* = 0.03, unadjusted), *Bifidobacterium catenulatum* (*P* = 0.03, unadjusted), and *Bifidobacterium longum* (*P* = 0.04, unadjusted).

The overall composition of the gut microbiota that increased or decreased after the prebiotic supplement compared to the placebo is shown in the circular cladograms (Supplementary Figures 2A,B). The only phylum that significantly changed after the prebiotic supplement was Actinobacteria. At the genus level (Supplementary Figure 2A), 11 genera significantly (*P* ≤ 0.05, unadjusted) changed with the prebiotic treatment compared to the placebo. The genera *Bifidobacterium, Anaerostipes*, and *Hungatella* increased, while the eight other genera decreased. At the species level (Supplementary Figure 2B), 22 species significantly (*P* ≤ 0.05, unadjusted) changed with the prebiotic treatment compared to the placebo. However, none of these changes remained statistically significant after correction for multiple testing.

### Gut Microbial Metagenome

**Figure 3:**
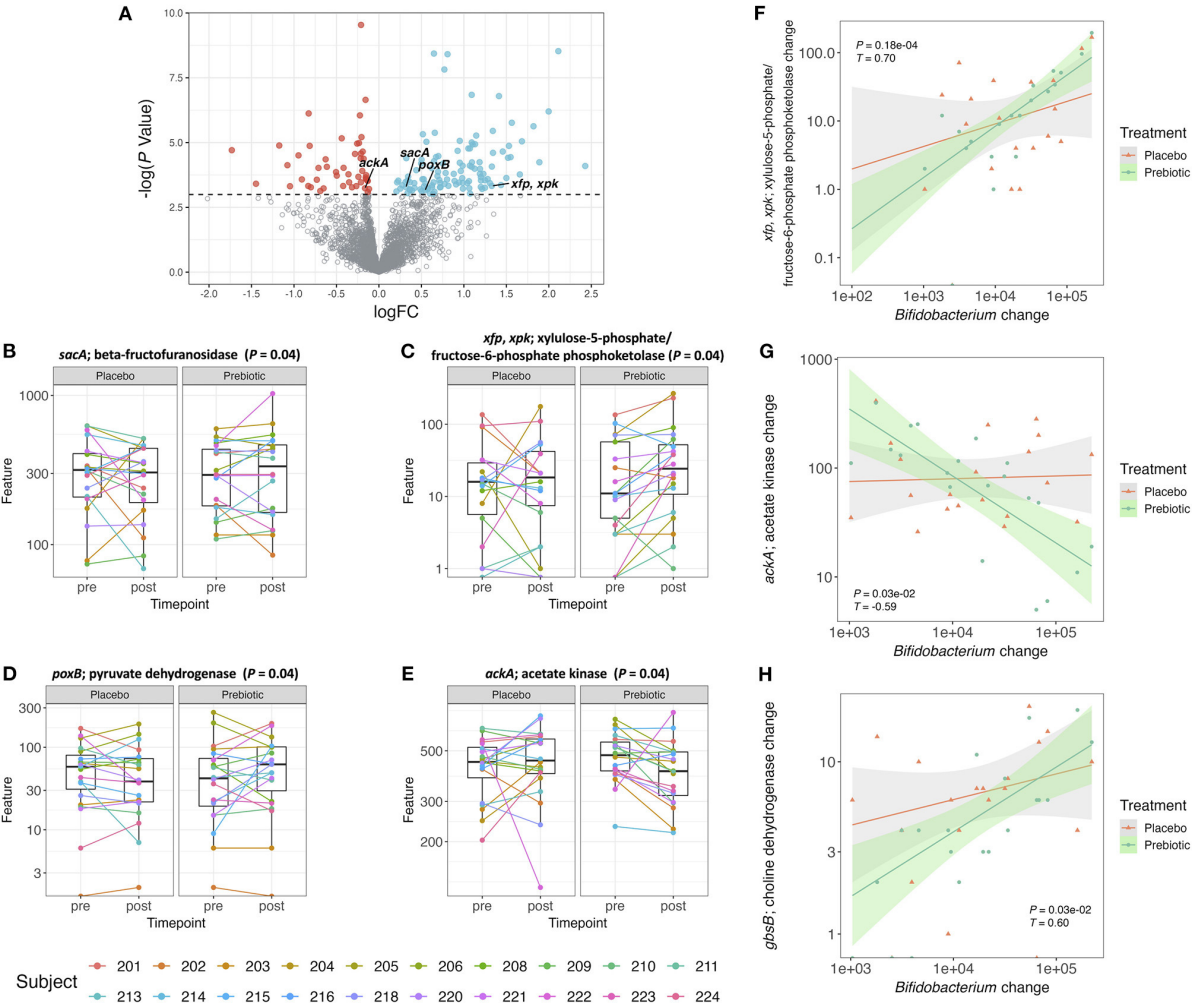
**(A)** Volcano plot of all detected genes. Genes with a logFC (post–pre treatment) > 0 and a –log(*P*-value) > 0.05 are colored blue and genes with a logFC <0 and a –log(*P*-value) > 0.05 are colored red. All the other genes are colored gray. **(B–E)** Box plot of gene counts pre- and post-treatment with placebo or prebiotic (*P* ≤ 0.05, unadjusted). **(F–H)** Correlation plot of changes (post–pre) in *Bifidobacterium* abundance against changes (post–pre) in gene counts for both placebo and prebiotic in 20 subjects (prebiotic: *P* ≤ 0.05, adjusted, Kendall *T*).

The abundance of several genes increased or decreased after the prebiotic supplementation compared to the placebo (Figure 3A). Overall, 163 out of 2,718 genes significantly (*P* ≤ 0.05, unadjusted) increased or decreased after the prebiotic arm. Among the genes that changed, 49 decreased and 114 increased with the prebiotic treatment.

The pathways associated with the bacterial genes were analyzed by gene set enrichment analysis (GSEA) (Supplementary Figure 3A). There were 10 pathways that were significantly (*P* ≤ 0.05, unadjusted) enriched by the prebiotic supplementation, however, these differences did not remain statistically significant after correction for multiple testing. Starch and sucrose metabolism and the pentose phosphate pathway were two enriched pathways associated with the genes that we specifically hypothesized to increase in their counts after the prebiotic supplement compared to the placebo. The gene involved in starch and sucrose metabolism that showed significant increase after the prebiotic treatment was *sacA* coding for beta-fructofuranosidase (Figure 3B). This enzyme produces fructose and glucose from sucrose (59). The gene associated with fructose utilization was found to be significantly increased after the prebiotic supplement which was mostly fructose-based sugars (Figure 3C). The genes *xfp, xpk*, encoding for xylulose-5-phosphate/fructose-6-phosphate phosphoketolase, were shown to be associated with the pentose phosphate pathway converting D-xylulose-5-phosphate to D-glyceraldehyde-3-phosphate as well as D-fructose 6-phosphate to D-erythrose-4-phosphate (60). The phosphoketolase reaction on xylulose-5-phosphate and fructose-6-phosphate is one of the major reactions found in the bifidobacteria shunt (“bifid shunt”) (61, 62). Bifidobacteria utilize the hexose sugars to generate ATP producing SCFAs as byproducts (63).

As certain bifidobacterial species are known for their ability to produce SCFA such as acetate and butyrate, we specifically hypothesized genes associated with the production of these SCFAs would increase in gene counts after the prebiotic supplement compared to the placebo. Pyruvate to acetate conversion is mediated by the gene *poxB* encoding for pyruvate dehydrogenase (64). Acetyl phosphate to acetate conversion is mediated by the gene *ackA* encoding for acetate kinase/phosphotransacetylase (64). Butyryl phosphate to butyrate conversion is mediated by the gene *buk* encoding for butyrate kinase (65). In this study, the gene, *poxB*, related to SCFA production, specifically acetate formation (66), was increased after the prebiotic supplement (Figure 3D). The *ackA* gene counts were shown to decrease after the prebiotic supplement compared to the placebo (Figure 3E). The gene counts of other genes (*pta, acs*, and *buk*) in bacterial SCFA production pathways did not change after the prebiotic supplementation (data not shown).

A treatment-stratified correlation analysis between changes (post–pre) in OTU counts of *Bifidobacterium* and changes (post–pre) in gene counts (Supplementary Figure 3B) was performed to ascertain if any gene abundance changes coincided with the increase in bifidobacterial abundance. The changes in bifidobacterial OTU counts at the genus level were significantly correlated with the changes in gene counts from the prebiotic arm but not with the placebo arm. Among 30 genes, 29 genes were positively correlated, and 1 gene was negatively correlated with change in *Bifidobacterium* (Supplementary Figure 3B). In the correlation analysis of change in *Bifidobacterium* with gene count change, the gene *sacA* was not significant after multiple testing correction (Supplementary Figure 3B). However, the correlations between changes in *Bifidobacterium* and changes in the gene counts of *xfp, xpk* did remain statistically significant after multiple testing correction (Figure 3F). A negative correlation between changes in *Bifidobacterium* and changes in *ackA* gene counts also remained statistically significant after multiple testing correction (Figure 3G). Additionally, the gene counts for *gbsB*, a choline dehydrogenase, increased, although not significantly (*P* = 0.07) after the prebiotic treatment compared to the placebo. The changes in the abundance of the *gbsB* gene was positively correlated with changes in *Bifidobacterium* (*P* < 0.05) (Figure 3H).

### Gut Microbe-Derived Metabolites

#### Plasma Metabolites

**Figure 4:**
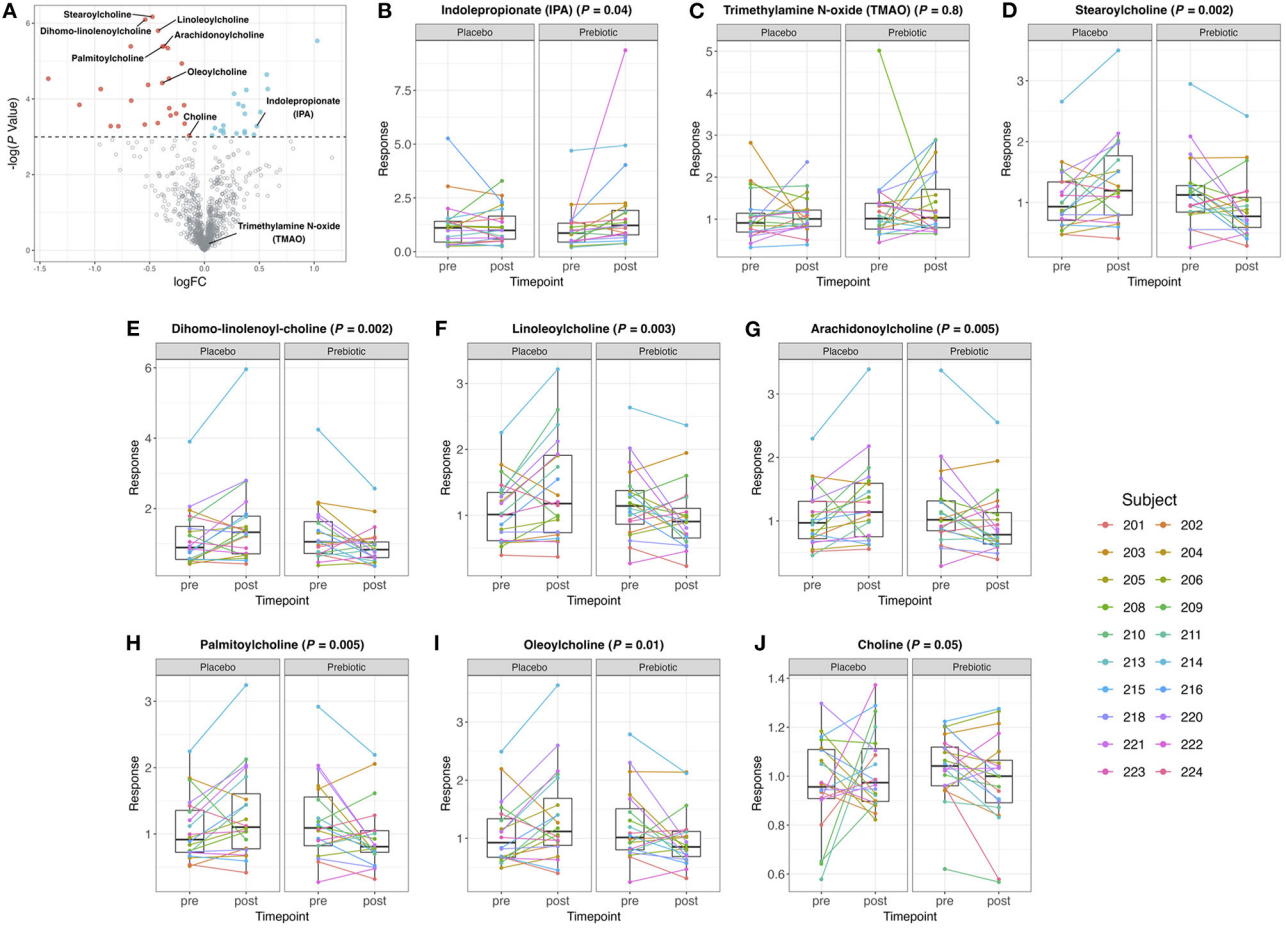
**(A)** Volcano plot of all metabolites in human plasma samples. Metabolites with a logFC > 0 and a –log(*P*-value) > 0.05 are colored blue and metabolites with a logFC <0 and a –log(*P*-value) > 0.05 are colored red. All the other metabolites are colored gray. **(B–J)** Box plots of IPA, TMAO, choline, and acylcholines concentrations pre- and post-treatment with placebo or prebiotic (unadjusted *P*-value).

Forty-five of 889 detected metabolites significantly (*P* ≤ 0.05, unadjusted) increased or decreased with prebiotic supplementation (Figure 4A). The results of differential expression analysis of each metabolite (IPA and TMAO) are shown in Figures 4B,C. The prebiotic supplement formulation increased the total amount of IPA (*P* = 0.04) compared to the placebo. However, the supplement had no significant effect on the total amount of TMAO (*P* = 0.84) compared to the placebo.

Significant decreases in choline and its acylated derivatives, the acylcholines were observed. Additionally, plasma concentrations of stearoylcholine, dihomo-linolenoyl-choline, linoleoylcholine, arachidonoylcholine, palmitoylcholine, oleoylcholine, and choline significantly decreased after the prebiotic intervention compared to the placebo (Figures 4D–J).

To determine whether any plasma metabolites were correlated with the abundance of *Bifidobacterium* a heatmap was generated to determine metabolites that are either positively or negatively associated with *Bifidobacterium* (Supplementary Figure 4A). Twenty-six metabolites were positively associated with *Bifidobacterium* and six metabolites were negatively associated with *Bifidobacterium* with adjusted *P*-values <0.05 after multiple testing correction. A total of six metabolites that significantly changed after the prebiotic treatment were also correlated with *Bifidobacterium* (Supplementary Figure 4A). Additionally, PLS-DA was performed to test if the prebiotic treatment had a discernible metabolomic signature. The prebiotic, while altering bifidobacterial abundance and increasing the concentration of IPA as well as decreasing the concentrations of several acylcholines, did not have an overall effect on the plasma metabolome, as shown by overlap of the time points in the scores plot (Supplementary Figure 4B).

#### Fecal Metabolites

SCFA concentrations in the stool samples were unchanged after the prebiotic treatment or placebo in healthy subjects (Supplementary Table 1).

## Discussion

Low intake of dietary fiber in adults is associated with a number of deleterious health effects including an increased risk for metabolic disease and inflammation (67–72). The recommended intake for dietary fiber is 14 g/1,000 kcal per day (73). In this study, a prebiotic fiber supplement that has been shown to affect microbial community structure and metabolites *in vitro* (unpublished), was utilized to determine the effects in human participants. The objective was to determine if a low dose (12 g) prebiotic fiber supplement can have a measurable impact on gut microbial community composition and human plasma metabolites in participants consuming low-fiber diets (<15 g/day).

The prebiotic supplement did not change the overall composition of the microbiome, and a high degree of inter-individual variability in the microbiome was observed, as demonstrated extensively in previous studies (74–79). However, consuming the prebiotic supplement for 4 weeks led to significant changes in the specific bifidobacterial species, as hypothesized, likely due to the composition of fructan oligosaccharides, which are known to act as a selective substrate for bifidobacteria. The prebiotic increased the relative abundance of the genus *Bifidobacterium* as well as that of several bifidobacterial species and the phylum Actinobacteria. *Bifidobacterium* species that increased included *B*. *bifidum, B. adolescentis, B*. *breve, B*. *catenulatum*, and *B*. *longum*. These results align with the results of other studies, which demonstrate increases in bifidobacteria after the consumption of fructan-based oligosaccharides (19, 21, 22, 80). Healthy adults who consumed as little as 5 g of inulin for 21 days showed significant increases in *Bifidobacterium* species including *B*. *adolescentis* and *B*. *bifidum* (81). Treatment with 16 g/day of inulin-type fructans for 3 months in obese women resulted in a significant increase in the species *B*. *adolescentis, B*. *pseudocatenulatum*, and *B*. *longum* (21). Two other studies supplementing inulin-type fructan prebiotics (15–16 g/d) increased *Bifidobacterium* but did not significantly alter the overall microbial community due to large inter-individual variability (36, 82).

In the current study, in addition to assessing the increase in relative abundance of bifidobacteria, we also sought to determine the gene functional changes at the level of the metagenome that occur in response to supplementation, to elucidate the specific mechanisms by which an increase in bifidobacterial abundance in the gut is associated with functional changes that may confer benefits to the host. We hypothesized that counts of genes that code for proteins involved in the transport and metabolism of the oligosaccharides contained within the prebiotic supplement would increase on the prebiotic arm. Specifically, we hypothesized that *sacA*, coding for the enzyme beta-fructofuranosidase, and *xfp, xpk*, coding for xylulose-5-phosphate/fructose-6-phosphate phosphoketolase, would both increase in response to the supplement. The beta-fructofuranosidase enzyme converts sucrose into glucose and fructose (59), which is involved in the utilization of glucose and fructose for growth or energy sources by bifidobacteria (83–86). We found that changes in OTU counts of *Bifidobacterium* and changes in abundance of the *sacA* gene were not significantly correlated. This is likely because many gut bacteria other than bifidobacteria also express beta-fructofuranosidase to utilize fructose as a substrate (87, 88), and thus the increase in the gene counts of this enzyme could not be attributed solely to the increase in bifidobacteria (84, 89). The enzymes xylulose-5-phosphate/fructose-6-phosphate phosphoketolase each convert D-xylulose-5-phosphate to D-glyceraldehyde-3-phosphate and D-fructose 6-phosphate to D-erythrose-4-phosphate (60). The dual enzymatic reaction of these phosphoketolases are related to bifidobacterial utilization of hexose sugars in the “bifid shunt”, producing acetyl phosphate as well as SCFAs as byproducts while generating ATP (62, 63). Bifidobacterial species are specifically known for their ability to produce acetate from fructooligosaccharides (90, 91). Under anaerobic conditions, such as the human gut, acetyl phosphate is converted to acetate, which is facilitated by acetate kinase encoded by the *ackA* gene (92). Another enzyme, pyruvate dehydrogenase, which produces acetate from pyruvate is encoded by the *poxB* gene (93). In the current study the gene counts of *ackA* decreased and those of *poxB* increased after the prebiotic treatment. This could result in an overall increase in the acetate pool, providing substrate for acetyl-CoA (94). The acetyl-CoA may in turn enter the TCA cycle for complete oxidation of sugar molecules in cellular respiration (95). Acetyl-CoA may also be involved in the production of intracellular butyrate (65, 96). However, gene counts of the *buk* gene associated with butyrate production and other genes related to the metabolic pathways of SCFA production did not change.

Furthermore, acetate, butyrate, and other SCFA concentrations in the stool samples were unchanged. It is not clear why the increase in acetate production genes was not associated with changes in acetate measured directly in fecal samples. The increases in these gene counts may not have been sufficient to be directly reflected in the plasma and fecal concentrations of acetate (97, 98). Similar results were found in clinical studies supplementing prebiotic (inulin-type fructans) in healthy individuals (36, 82). The utilization of acetate and butyrate by the host as energy sources, particularly in the colon (99, 100), may be one of the contributing factors for the lack of measurable effects on acetate and butyrate concentrations. Alternatively, the prebiotic supplement at a dosage of 12 g/day may not be sufficient to produce detectable changes in fecal SCFA concentrations. Lastly, discrepancies in the water content of each fecal sample may have decreased the signal to noise ratio. A recent paper demonstrated that lyophilization of fecal samples reduces detection errors from water content and improves SCFA stability (101). Thus, implementing fecal lyophilization may be able to capture more accurate SCFA concentrations in future studies.

In this study we sought to further elucidate the relationships between gene count changes and the increase in bifidobacterial abundance by performing a treatment-stratified correlation analysis between the change in the OTU counts of the genus *Bifidobacterium* and changes in counts of genes. Two genes, *xfp, xpk* and *ackA*, were found to have a positive and a negative association with *Bifidobacterium*, respectively. These genes were also differentially expressed after the prebiotic supplement compared to the placebo. The genes *xfp, xpk* are related to a unique carbohydrate metabolism pathway utilizing the phosphoketolase enzyme in bifidobacteria, known as the bifid shunt (102). The positive correlation between changes in bifidobacterial OTU counts and changes in counts of *xfp, xpk* genes may be due to the unique utilization of the prebiotic supplement during the intervention. The *ackA*-*pta* pathway and *poxB* pathway are known to have an important role during the exponential growth phase and stationary phase, respectively, in *E. coli* for producing acetate (64). In *E. coli*, a small RNA, SdhX, regulates encoding enzymes of the TCA cycle and represses the expression of *ackA* while *pta* is not significantly affected (103). Several papers show significance of the acetate fermentation genes *ackA*-*pta* in bifidobacteria (103–105).

An interesting gene shown to be positively correlated with bifidobacteria was *gbsB* encoding for choline dehydrogenase. Not much is known about the role of choline dehydrogenase in bifidobacteria, and this may be the first report of a positive correlation between *gbsB* gene and *Bifidobacterium*. A paper showed decreased levels of choline in rats treated with certain strains of *Bifidobacterium* and *Lactobacillus* (106). The most well-studied links between choline and gut microbiota are associated with trimethylamine (TMA) production (107–109). The *cut* gene cluster, including choline-TMA lyase, encoded by the *cutC* gene is expressed by diverse taxa of Firmicutes and Proteobacteria converting choline into TMA (110). Prebiotic supplementation may modulate the gut microbiota that utilize choline and thus affect plasma TMA or TMAO concentrations, as has been shown in obese children (111) and in mice (112). However, the concentration of TMAO did not change after the prebiotic compared to the placebo, confirming a similar lack of effect in other studies (35, 38). On the other hand, choline metabolites were found to uniformly decrease after the prebiotic treatment including choline itself and several acylcholines including stearoylcholine, dihomo-linolenoyl-choline, linoleoylcholine, arachidonoylcholine, palmitoylcholine, and oleoylcholine. In colon cancer patients the consumption of rice bran results in a decrease in palmitoylcholine, linoleoylcholine, and oleoylcholine (113). In contrast, infants supplemented with rice bran showed increases in palmitoylcholine, oleoylcholine, linoleoylcholine, and stearoylcholine (114). Long-chain acylcholines have been found to have associations with a number of disease states (115), including elevated concentrations in endometrial cancer patients (116) and patients at high risk of pulmonary embolism (117), and lower concentrations in patients with myalgic encephalomyelitis/chronic fatigue syndrome (118) and chronic thromboembolic pulmonary hypertension (119). Acylcholines are known to have cholinergic signaling properties, with important implications for signaling through the nicotinic acetylcholine receptor (α7 nAChR), and effects on cytokine synthesis in macrophages and T cells (115, 120).

In addition, we hypothesized that the metabolite IPA would increase after the prebiotic intervention, and indeed, an increase in IPA was observed. Previous studies have shown that the concentration of IPA in the blood was positively correlated with dietary fiber intake (121). A clinical study in healthy individuals who consumed a high-fiber Mediterranean diet also showed an increase in IPA (38). IPA is produced by gut microbes from tryptophan and has been determined to play a crucial role in sustaining mucosal barrier function (23, 122, 123). Evidence on the beneficial health effects of IPA is growing (124–126), highlighting the therapeutic potential of probiotics and prebiotics that increase IPA production.

In this tightly controlled dietary intervention study, great care was taken to maximize the stability of the background diet as much as possible in order to maximize the ability to detect an effect of the prebiotic supplement. Participants were instructed to maintain their habitual diet throughout the course of the 12-week study protocol. We confirmed through 3-day diet record analysis that there were no significant changes in the background diet. This intervention in young, healthy participants who consume a low-fiber diet, but who were nonetheless metabolically healthy, did not affect cardiometabolic profiles. Fasting glucose, insulin, and lipid panels were not significantly altered after the prebiotic supplementation compared to the placebo. Several studies show clinically meaningful decreases in fasting or postprandial blood glucose and insulin concentrations in response to fiber supplementation (1, 127, 128). However, many of these studies were conducted in participants with elevated baseline values of these cardiometabolic parameters, such as individuals with metabolic syndrome (127) or type 2 diabetes (129), or in healthy participants (130, 131) but at much higher fiber doses (e.g., 38 vs. 12 g/d in this study) (1). Other studies show results that are in line with this study demonstrating no significant differences in cardiometabolic profiles between groups supplemented with fiber diet vs. control diet (132, 133).

The prebiotic supplement did not have gender-specific differences on the overall gut microbial composition and diversity as well as other secondary outcomes. The prebiotic supplement was well-tolerated by the participants without any abdominal discomfort reported during the study period. The strength of this study is in the study design, which was randomized, placebo-controlled, double-blind, crossover. A weakness of this study is the relatively small number of subjects and relatively short intervention period. However, a crossover design of the study increases the power to detect changes in response to the treatment with a small number of subjects and a shorter period. Additionally, several studies showed even a short-term intervention could result in a distinct change in the gut microbiome (36, 134–137). A previous study showed a significant increase in bifidobacteria after daily consumption of a mean intake of inulin-type fructans (15 g) in 26 healthy individuals in just 2 weeks (36). In this study, a 4-week intervention period was additionally chosen because it would allow for sampling at the same phase of the menstrual cycle for the female participants, eliminating the potential confounding effects of hormonal fluctuations on the gut microbiome.

Taking into account the fact that the prebiotic was hypothesized to be tested as a daily supplement to healthy individuals, the results that we found may be beneficial to the general population and suggest a simple way to increase the relative abundance of bifidobacteria, which is well-known for its beneficial health effects. On the other hand, the population of bifidobacteria not only decreases as we age but becomes absent in the gut of patients with certain diseases. Thus, maintaining the relative abundance of bifidobacteria throughout the life span may be crucial even in healthy individuals. However, other beneficial health effects of consuming prebiotics (e.g., reductions in triglycerides, LDL-cholesterol) were not shown in this study, which is likely due to the fact that the participants were already healthy at the start of the study.

In conclusion, supplementation with 12 g/day of a diverse, prebiotic dietary fiber blend resulted in measurable increases in beneficial *Bifidobacterium* species, changes in counts of genes associated with the utilization of the prebiotic as well as acetate production, and changes in plasma IPA, choline, and acylcholines in generally healthy individuals who consume a low-fiber diet. These results demonstrate a tangible benefit of a relatively low dose of a prebiotic fiber supplement in individuals who do not consume recommended amounts of dietary fiber, highlighting that even small, easy to incorporate changes in dietary intake can have beneficial effects on gut microbiome-mediated metabolism.

## Data Availability Statement

The datasets presented in this study can be found in online repositories. The names of the repository/repositories and accession number(s) can be found below: ENA; PRJEB52881.

## Ethics Statement

The studies involving human participants were reviewed and approved by Institutional Review Board of University of California, Davis. The patients/participants provided their written informed consent to participate in this study.

## Author Contributions

JK, CW, MB, RB, RM, and AZ conceived and designed the study. JK, JZ, and JA conducted the clinical trial. JK and XT processed and analyzed the data. JK wrote the manuscript and created figures and tables. JK, XT, CW, MB, RB, RM, JZ, JA, and AZ contributed to editing and reviewing the manuscript. All authors have read and agreed to the published version of the manuscript.

## Funding

This work was supported by the USANA Health Sciences Inc.

## Conflict of Interest

JK and AZ have received research support from USANA Health Sciences, Inc. CW, MB, RB, and RM are employees of USANA Health Sciences, Inc. These interests have been reviewed and managed by the University of California, Davis in accordance with its Conflict-of-Interest policies. The remaining authors declare that the research was conducted in the absence of any commercial or financial relationships that could be construed as a potential conflict of interest. The authors declare that this study received funding from USANA Health Sciences, Inc. The funder had the following involvement in the study: study conceptualization and manuscript review.

## Publisher's Note

All claims expressed in this article are solely those of the authors and do not necessarily represent those of their affiliated organizations, or those of the publisher, the editors and the reviewers. Any product that may be evaluated in this article, or claim that may be made by its manufacturer, is not guaranteed or endorsed by the publisher.
