# Supplementary material for: Multi-Omic Analyses Reveal Bifidogenic Effect and Metabolomic Shifts in Healthy Human Cohort Supplemented With a Prebiotic Dietary Fiber Blend
Source: Front Nutr. 2022 Jun 17;9:908534. doi: 10.3389/fnut.2022.908534 (PMC9248813; doi:10.3389/fnut.2022.908534)
Supplement: Supplementary file 1 [file Table_1.DOCX]

Supplementary Material

# Supplementary Figures and Tables

## Supplementary Figures


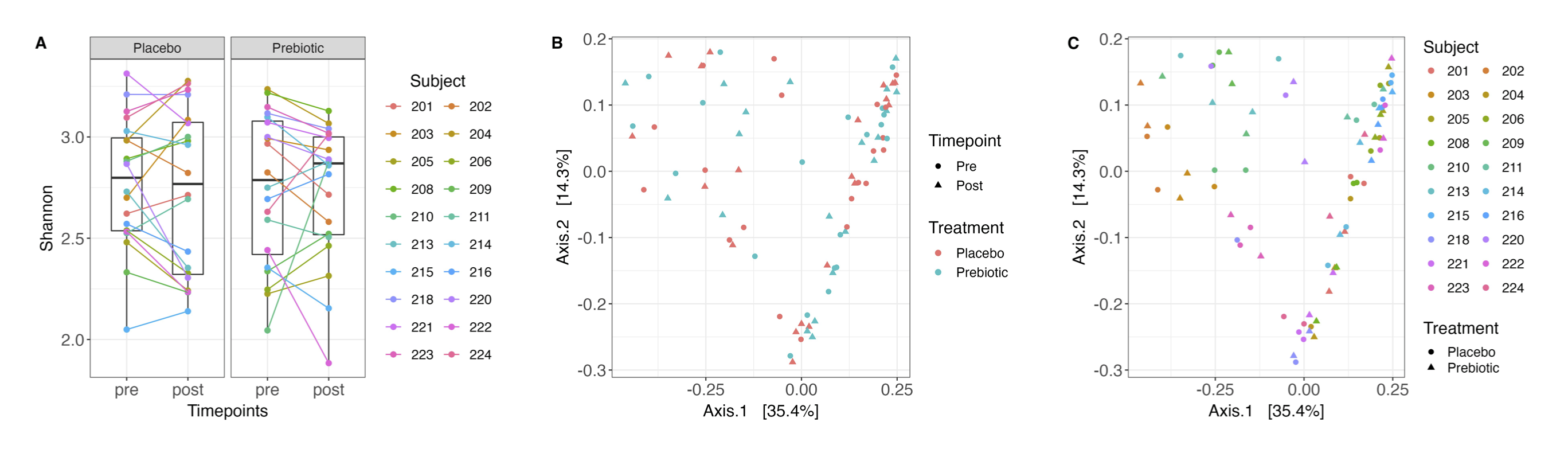


**Supplementary Figure 1.** **(A)** Shannon diversity (*P* = 0.75) of the gut microbiome community pre- and post-treatment with placebo or prebiotic. Bray–Curtis dissimilarity of the gut microbiome community color coded by **(B)** treatment and **(C)** subject, respectively.


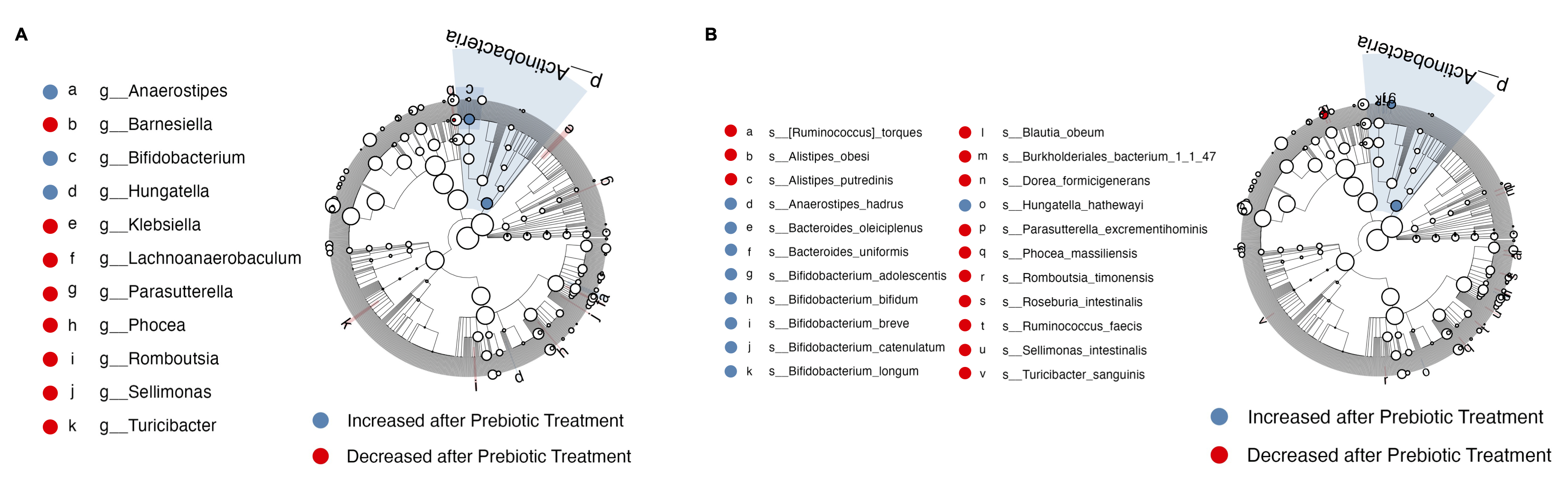


**Supplementary Figure 2.** Circular cladograms of the gut microbiota that significantly (*P* ≤ 0.05, unadjusted) changed after the prebiotic at genus **(A)** and species **(B)** levels. The blue dots indicate microbes that increased, and the red dots indicate microbes that decreased after the prebiotic treatment.


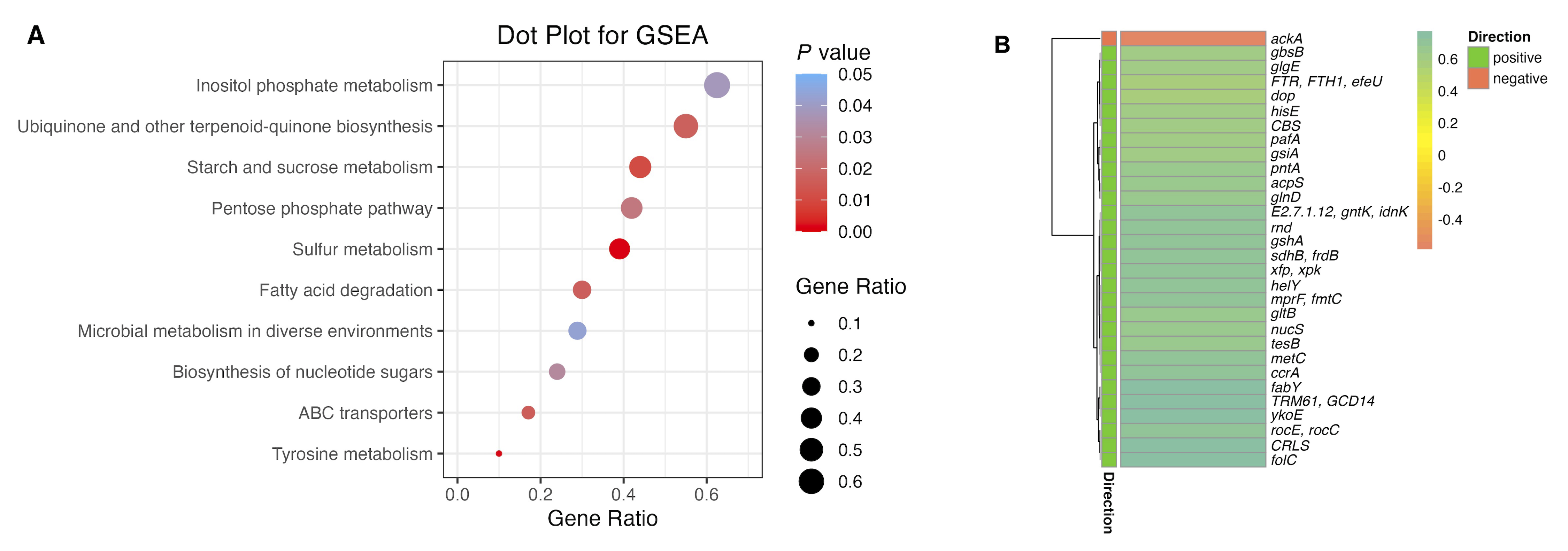


**Supplementary Figure 3.** **(A)** Dot plot of gene set enrichment analysis. Metabolic pathways enriched by the prebiotic treatment compared to the placebo were calculated and indicated by the color of *P* value (unadjusted) and size of Gene Ratio. The plot displays the Gene Ratio (number of significant genes related to KEGG pathway / total number of significant genes) by the size of dots. **(B)** Heatmap of genes that were positively (green) or negatively (pink) associated with *Bifidobacterium*. The scale represents Kendall’s correlation coefficient. The adjusted *P* values of 30 genes are less than 0.05 after Benjamini–Hochberg adjustment.


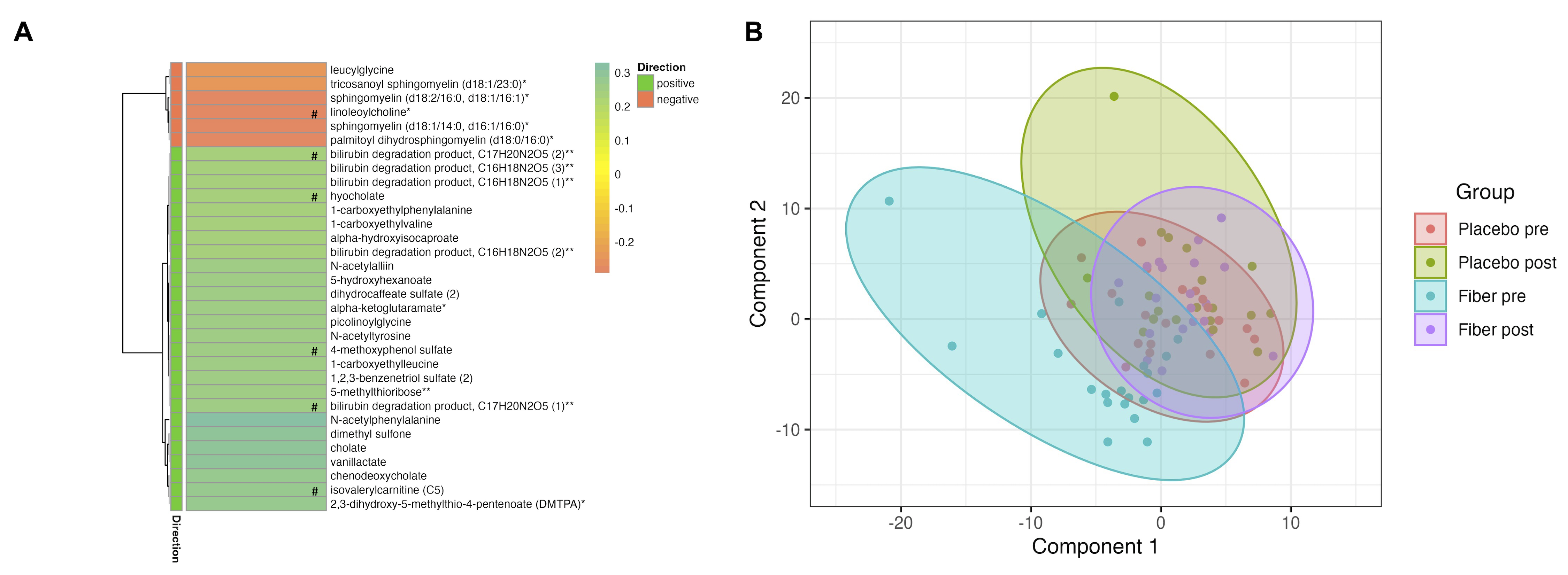


**Supplementary Figure 4.** **(A)** Heatmap of metabolomic profiles that were positively (green) or negatively (pink) associated with *Bifidobacterium*. The scale represents Kendall’s correlation coefficient. The adjusted *P* values of 32 metabolites are less than 0.05 after Benjamini–Hochberg adjustment (*suspected metabolites from mass analysis, ^#^metabolites that were found in both volcano plot and heatmap). **(B)** PLS-DA score plot generated from each treatment and timepoint groups.

| **Supplementary Table 1.** Fecal SCFA concentrations.* | | | | |
| --- | --- | --- | --- | --- |
| SCFA | logFC | Average expression | t | *P* value |
| Acetic Acid | 0.011 | 2.29 | 0.068 | 0.946 |
| Propionic Acid | 0.505 | 0.337 | 1.24 | 0.219 |
| Isobutyric Acid | -0.180 | -1.39 | -0.634 | 0.529 |
| Butyric Acid | 0.065 | 0.869 | 0.334 | 0.740 |
| Isovaleric Acid | 0.177 | -0.510 | 0.836 | 0.406 |
| Valeric Acid | 0.109 | -0.527 | 0.575 | 0.568 |
| Hexanoic Acid | 0.087 | -1.48 | 0.391 | 0.697 |
| *Data are presented as comparison between post−pre prebiotic fiber vs placebo in log fold change (logFC), average expression across all samples, logFC divided by its standard error (t), and *P* values for each SCFA with a differential expression analysis. | | | | |
